# Vitamin D in Type 2 Diabetes Mellitus According to Diabetic Kidney Disease Stage: What Is the Target in Clinical Practice?

**DOI:** 10.3390/nu18091408

**Published:** 2026-04-29

**Authors:** João Soares Felício, Lícia Oliveira Ruivo, Inessa Silveira Bezerra, Maria Eduarda Nunes Avelar do Carmo, Isabel Jacob Fernandes, Beatriz Dias Lobo, Lilian de Souza D’Albuquerque Silva, Franciane Trindade Cunha de Melo, Ana Carolina Contente Braga de Souza, Natércia Neves Marques De Queiroz, Márcia Costa dos Santos, Lorena Vilhena De Moraes, Mayana Batista Barros, Alan Pinheiro Fernandes, Paula Gabriella Costa da Penha, Ana Clara Fleury da Fonseca Rodrigues, Pedro Paulo Freire Piani, Ana Regina Bastos Motta, Valéria Suênya Galvão Leal, Melissa de Sá Oliveira dos Reis, Alyne Maciel Lobato, Karem Mileo Felício, Priscila Boaventura Barbosa De Figueiredo

**Affiliations:** Endocrinology Department, University Hospital João de Barros Barreto, Federal University of Pará, Belem 66073-005, Brazil; liciaruivomed@gmail.com (L.O.R.); inessasilbez@gmail.com (I.S.B.); eduardaavelar05@gmail.com (M.E.N.A.d.C.); isabel.fernandes@ics.ufpa.br (I.J.F.); beatrizxlob@gmail.com (B.D.L.); liliandalbuquerque@yahoo.com.br (L.d.S.D.S.); franciane.melo@hotmail.com (F.T.C.d.M.); carol.souza25@hotmail.com (A.C.C.B.d.S.); natercianeves@hotmail.com (N.N.M.D.Q.); marciacsantos29@gmail.com (M.C.d.S.); dramayanabarros@gmail.com (M.B.B.); alanfernandesendocrino@gmail.com (A.P.F.); paula.penha@hotmail.com (P.G.C.d.P.); anafleuryy@gmail.com (A.C.F.d.F.R.); pedropiani@yahoo.com.br (P.P.F.P.); arbmotta@gmail.com (A.R.B.M.); valeriasuenya@gmail.com (V.S.G.L.); melissabiomedica1@gmail.com (M.d.S.O.d.R.); alynelobato94@gmail.com (A.M.L.); karemfelicio@yahoo.com.br (K.M.F.); pribfigueiredo@gmail.com (P.B.B.D.F.)

**Keywords:** diabetes mellitus, type 2 diabetes mellitus, diabetic kidney disease, vitamin D, vitamin D standards

## Abstract

**Background/Objectives:** Low vitamin D (VD) has been associated with diabetic kidney disease (DKD). Nevertheless, VD normality concentrations remain under discussion. **Objectives:** To evaluate VD concentrations in early and advanced DKD stages according to the Endocrine Society and Institute of Medicine standards in patients with type 2 diabetes mellitus (T2DM). **Methods:** A cross-sectional study included 80 patients with T2DM and DKD in early (urine albumin–creatinine ratio (UACR) between 30 and 299 mg/g), group 1 (*n* = 29), and advanced stage (UACR ≥ 300 mg/g), group 2 (*n* = 51). **Results:** Compared to group 1, patients in group 2 showed lower 25(OH)D concentrations (26.7 ± 10.1 vs. 30.2 ± 8.0 ng/mL; *p* ≤ 0.05). In group 2, when the serum VD concentrations were stratified according to the Endocrine Society, VD concentrations ≥ 30 ng/mL were associated with a considerably reduction in UACR compared with VD < 20 ng/mL (365.9 ± 268.3 vs. 937.6 ± 634 mg/g; *p* < 0.05), but not when compared to the range of 20–29.9 ng/mL (739.8 ± 1138 vs. 937.6 ± 634 mg/g; *p* = NS). Additionally, in this group, our regression model showed that each unit increase in VD was associated with a UACR reduction of 26.1 mg/g (R^2^ = 0.103; *p* < 0.05). **Conclusions:** Higher vitamin D concentrations were associated with improved UACR only in clinical DKD, particularly when VD was at least 30 ng/mL.

## 1. Introduction

Diabetic kidney disease (DKD) represents one of the major microvascular complications of diabetes mellitus (DM) and is associated with increased morbidity and mortality [1,2,3], being the leading cause of kidney failure requiring dialysis or kidney transplantation worldwide [4]. In parallel, several cross-sectional studies have associated low vitamin D (VD) concentrations with increased kidney damage, particularly with a rise in urine albumin-to-creatinine ratio (UACR) [5,6,7,8,9,10,11,12]. A nephroprotective effect has also been suggested following VD supplementation [13,14,15,16,17,18,19]. Nevertheless, the normal reference concentrations of VD in the general population remain controversial [18,19,20,21].

In patients with diabetes mellitus, nephroprotective effects have already been described for VD, possibly mediated by mechanisms associated with the renin–angiotensin system as well as reductions in inflammatory mediators and fibrosis [22,23,24,25]. In addition, DKD and the consequent reduction in glomerular filtration rate (GFR) have been reported to be related to lower VD concentrations, a relationship that has been described in terms of reduced renal production capacity, impaired reabsorption, and diminished bioavailability, uptake, and conversion of both active and inactive VD forms [23,26,27].

Finally, patients with type 2 diabetes mellitus (T2DM) at different stages of kidney disease have not been described within the same population. These data could be useful for identifying the VD concentrations most strongly associated with nephroprotection, thereby guiding posterior VD supplementation strategies in patients with T2DM and DKD.

## 2. Materials and Methods

### 2.1. Study Design and Patients

This cross-sectional study aimed to evaluate the association between VD concentrations and kidney damage in patients diagnosed with T2DM across different stages of DKD. No drug intervention was performed in these individuals, and no patient used VD or calcium supplements within the three months preceding screening and during the study period. Eighty patients with T2DM and DKD were recruited from the Endocrinology Department of the João de Barros Barreto University Hospital between 2023 and 2025. Patients were classified in two distinct stages of DKD: 29 in the early stage (UACR: 30 to 299 mg/g—group 1—corresponding to stage A2 chronic kidney disease (CKD) (KDIGO)), and 51 in the advanced stage (UACR ≥ 300 mg/g—group 2—corresponding to stage A3 CKD (KDIGO) (2)) of DKD. All procedures was conducted in accordance with the Declaration of Helsinki and the Nuremberg Code of 1975 (revised in 2008). The study was approved on 5 June 2023 by the Ethics Committee of João de Barros Barreto University Hospital with reference number 88974918.6.0000.0017. An informed consent form (ICF) was obtained from all patients.

The inclusion criteria were: (a) provision of ICF obtained prior to any study procedure; (b) patients with T2DM over 30 years of age with regular follow-up with an endocrinologist; (c) treatment with oral hypoglycemic agents or insulin at a stable dose for at least three months; (d) presence of UACR ≥ 30 mg/g; (e) GFR between >25 and <90 mL/min/1.73 m^2^); (f) patients using ACE (angiotensin-converting enzyme) or ARBs (angiotensin receptor blockers) were required to be on a stable dose for at least four weeks prior to study enrollment; (g) ability and willingness to perform all study procedures. The exclusion criteria included: (a) diabetes types other than type 2, such as type 1 diabetes; (b) prior history of disorders affecting bone metabolism; (c) history of hepatic disease; (d) use of vitamin D or calcium supplements within the three months preceding screening; (e) uncontrolled hyperthyroidism or hypothyroidism; (f) women who were pregnant, planning to become pregnant, or breastfeeding; (g) comorbid conditions considered by the investigator to potentially impact life expectancy; and (h) alcohol abuse or recreational drug use that, in the investigator’s judgment, could compromise participant safety or the study procedures.

All patients received physical activity and dietary guidance in accordance with the American Diabetes Association (ADA) guidelines [28], in addition to follow-up at the Endocrinology Department.

### 2.2. Data Collection

After applying the eligibility criteria, meeting all inclusion criteria and none of the exclusion criteria, patients were considered eligible to begin the research procedures. The diagnosis of T2DM was established through clinical and laboratory procedures [29].

Data collection was performed during scheduled appointments and consisted of reviewing medical records (pre-existing clinical conditions, demographic data, insulin use, and other current medications), physical examinations, and laboratory tests through the collection of blood and urine samples. DKD was assessed according to the guidelines of the American Diabetes Association and Kidney Disease: Improving Global Outcomes (KDIGO) [2,30].

Serum 25-hydroxyvitamin D (25(OH)D) concentrations were determined using the DiaSorin LIAISON 25-OH-Vitamin D TOTAL chemiluminescence immunoassay (DiaSorin, Stillwater, MN, USA) [31]. This method belongs to the group of techniques assessed by the Vitamin D External Quality Assessment Service (DEQAS), the largest specialized external quality control (proficiency testing) program dedicated to the measurement of vitamin D metabolites including 25(OH)D and 1,25(OH)_2_D. Glycated hemoglobin (HbA1C) was performed using high-performance liquid chromatography (HPLC) [32]. Fasting plasma glucose, total calcium, total cholesterol and fractions (low-density lipoprotein (LDL) and high-density lipoprotein (HDL)), and triglycerides were analyzed using automated colorimetric methods. Albuminuria was evaluated by immunoturbidimetry [33]. GFR was estimated using the CKD-EPI equation, refitted without race variables [34]. UACR was assessed in three separate spot urine samples.

### 2.3. Classification of DKD

UACR was measured three times in all patients, at different time points. Three categories were considered: normal UACR (<30 mg/g), early stage (UACR 30–299 mg/g), and advanced stage (UACR ≥ 300 mg/g) of DKD [2].

For clarity and consistency throughout the manuscript, patients with moderately increased and severely increased albuminuria were classified as having early and advanced stages of DKD, respectively.

### 2.4. Vitamin D Assessment

For VD assessment, two relevant guidelines were used: The Institute of Medicine (IOM) [20], which classifies 25(OH)D concentrations as deficient (<20 ng/mL) and normal (≥20 ng/mL), and the Endocrine Society guidelines [21], which classifies serum 25(OH)D concentrations as deficient (<20 ng/mL), insufficient (20–29.9 ng/mL), and sufficient (≥30 ng/mL).

### 2.5. Statistical Analysis

Data were organized and analyzed using Sigma Stat version 3.5 (Jandel Scientific Corporation, Chicago, IL, USA) and the Statistical Package for the Social Sciences (SPSS) version 22 (International Business Machines Corporation, Chicago, IL, USA).

Statistical analyses were conducted according to data distribution, with normality assessed using the Shapiro–Wilk test (*p* > 0.05). A two-tailed *p*-value < 0.05 was considered statistically significant in all analyses. Categorical variables were expressed as absolute numbers and frequencies (%). Continuous variables with normal distribution were presented as mean ± standard deviation. Comparisons between two groups for normally distributed variables were performed using the Student’s *t*-test, whereas one-way analysis of variance (ANOVA) was applied for comparisons involving more than two groups. Pearson’s correlation coefficient was used to assess associations between normally distributed variables. For non-normally distributed variables, the Mann–Whitney U test was used for comparisons between two groups, and the Kruskal–Wallis test for comparisons among more than two groups. Associations between categorical variables were evaluated using the chi-square test or Fisher’s exact test, as appropriate. A linear regression analysis was performed with UACR as the dependent variable and serum 25(OH)D concentration as the independent variable. Additionally, a multivariable logistic regression analysis was performed to adjust for potential confounding variables (age, sex, duration of diabetes, HbA1c, BMI, lipid profile, use of angiotensin-converting enzyme inhibitors (ACEis) and angiotensin II receptor blockers (ARBs), sodium-glucose cotransporter-2 (SGLT2) inhibitors, beta-blockers, and glucagon-like peptide-1 (GLP-1) agonists) using UACR values as the dependent variable and VD concentration as the independent. In this model, UACR values were used as the dependent variable, and serum VD concentration was included as the independent variable. The expected power to comparisons was >0.8, considering the differences between VD concentrations and UACR.

The sample size was estimated first to detect a difference in VD concentration between different stages of DKD using the *t*-test, considering an expected mean difference of 4 ng/mL, an expected standard deviation of 8 ng/mL, and a desired power of 0.8, with *p* < 0.05. This calculation indicated a minimum sample size of 64 individuals. In addition, a sample size of 70 individuals was estimated to detect a correlation between UACR values and VD concentrations, considering a desired power of 0.8, *p* < 0.05, and a minimum correlation coefficient of 0.3.

## 3. Results

Table 1 includes the demographic and clinical parameters of all patients. As expected, group 2 showed a longer duration of T2DM and a higher prevalence of diabetes complications, notably diabetic neuropathy, when compared to group 1.

The laboratory parameters of both groups are presented in Table 2. Consistent with renal impairment, group 2 showed higher serum creatinine, lower GFR, higher UACR, and lower 25(OH)D concentrations. Furthermore, group 2 also showed worse lipid profile parameter and higher fasting plasma glucose (FPG) levels.

Laboratory parameters were compared according to VD classification criteria (Table 3—IOM and Table 4—Endocrine Society). Only in group 2 did the UACR values show significant differences across VD concentrations, considering the IOM and Endocrine Society criteria. When serum 25(OH)D was stratified according to the Endocrine Society criteria, VD concentrations ≥ 30 ng/mL were associated with a considerably reduction in UACR when compared with VD < 20 ng/mL, but no significant difference was observed in the range of 20–29.9 ng/mL. These findings indicate that higher VD concentrations are associated with lower UACR levels in patients with advanced DKD, particularly when VD concentrations are ≥30 ng/mL, a threshold previously recommended by the Endocrine Society for the general population.

The distribution of serum 25(OH)D concentrations according to UACR tertiles is shown in Table 5. When VD concentrations were analyzed according to UACR tertiles, no significant differences were observed in group 1. In group 2, the serum 25(OH)D concentrations differed significantly across UACR tertiles, with progressively lower vitamin D concentrations observed in the higher UACR tertiles.

To further explore this association, a linear regression analysis was conducted. The model indicated that each unit increase in VD concentration was associated with an approximate reduction of 26.1 mg/g in UACR, exclusively in group 2 (R^2^ = 0.103; *p* < 0.05).

Additionally, a multiple logistic regression analysis was performed to adjust for potential confounding variables. UACR values were used as the dependent variable, and VD concentration was included as the dependent variable. The model demonstrated that VD deficiency may predict worse UACR values, was independently associated with worse UACR status, regardless of age, sex, duration of diabetes, HbA1c, BMI, lipid profile, use of ACEi/ARBs, use of SGLT2 inhibitors or GLP-1 receptor agonists, and beta-blockers (R^2^ = 0.303; *p* < 0.05).

## 4. Discussion

Our data demonstrated an association between higher VD concentrations and reduced UACR only in individuals with T2DM and advanced-stage DKD, particularly when 25(OH)D concentrations were at least 30 ng/mL. This pattern was consistent when the 25(OH)D concentrations were analyzed across the UACR tertiles, with increasing UACR occurring in parallel with worsening VD status. In addition, regression analysis showed that, exclusively in group 2, each 1 ng/mL decrease in VD concentration was associated with an approximate increase of 26.1 mg/g in UACR. Notably, this association was independent of glycated hemoglobin levels. To our knowledge, this is the first study to investigate the relationship between VD concentrations and renal excretion parameters across different stages of DKD in the same population with T2DM, comparing the difference in VD concentrations between two distinct clinical guidelines.

The most recent Endocrine Society guidelines no longer define a single target threshold for VD concentrations in healthy adults and therefore does not recommend routine measurement of serum VD in the general population under 75 years of age [35]. Regarding supplementation for preventive purposes, recommendations are restricted to specific groups, including children and adolescents (10–18 years), pregnant women, adults older than 75 years, and individuals with prediabetes. Independent investigations have shown divergent interpretations of the Endocrine Society recommendations, with some studies (primarily clinical trials involving cardiovascular and bone-mineral outcomes) supporting these recommendations [36,37,38,39,40,41,42], while others oppose the guidelines (basically about metabolic, renal, inflammatory and neurologic outcomes) [19,43,44,45,46,47]. Recently, our group suggested a VD cutoff of 26 ng/mL for subclinical hyperparathyroidism (using data from a population-based study including 31,853 individuals) [48]. These findings are consistent with those reported by Jaedy at al., who performed a meta-analysis with 16 cohort studies and reported a beneficial effect of VD at 30 ng/mL concentrations, whereas concentrations <25 ng/mL and >35 ng/mL were associated with increased mortality risk [49].

Considering populations with chronic kidney disease (CKD), serum VD sufficiency thresholds remain heterogeneous across clinical guidelines [2,50,51,52]. The KDIGO guidelines for diabetes management in CKD (2022) and for CKD–mineral and bone disorder do not endorse a universal target concentration for 25(OH)D, but acknowledge the high prevalence of vitamin D deficiency in CKD and recommend the assessment and correction of deficiency according to strategies used in the general population [2,50]. In contrast, European consensus statements explicitly consider serum 25(OH)D concentrations ≥ 30 ng/mL as indicative of sufficiency, emphasizing this threshold as clinically relevant for skeletal health in both pediatric and adult CKD populations [51]. Recent studies in CKD and kidney transplantation acknowledge discrepancies across VD recommendations, while evidence for renal or metabolic benefits, particularly in DKD, remains limited [53,54,55,56,57,58].

Studies evaluating the ideal level of VD in patients with DKD are limited [5,6,7,8,9,10]. Cross-sectional and retrospective studies in predominantly T2DM populations have explored the association between DKD and vitamin D status, with consistent results [5,6,7,8,9,10]. Shahwan et al. reported a high prevalence of VD deficiency among 453 patients with T2DM and DKD, particularly in older individuals [5]. Similarly, Zomorodian et al. reported a higher prevalence of macroalbuminuria in patients with T2DM and VD < 20 ng/mL [8]. Diaz et al., analyzing 1216 patients with diabetes, demonstrated that lower VD concentrations were associated with a higher risk of DKD [9]. Likewise, De Boer et al. described an association between lower VD concentrations and an increased risk of microalbuminuria in patients with type 1 diabetes mellitus (T1DM) [10]. Finally, our group has published two cross-sectional studies that investigated this issue in T2DM and T1DM [11,12]. In the VIDAMAZON study, we analyzed data from 1576 patients and found a clear association between low VD concentrations and increasing UACR, independently of glycemic control [11].

Furthermore, evidence from longitudinal studies has expanded upon cross-sectional findings, supporting an association between low VD concentrations and DKD [13,14,15,16,17]. De Zeeuw et al. conducted a study involving 281 patients with stages 2 to 4 DKD to evaluate the anti-albuminuric effects of paricalcitol, observing reductions in 24-h albuminuria, GFR, and blood pressure [13]. Esfandiari et al. analyzed the effects of weekly supplementation with 50,000 IU of vitamin D_3_ in 50 patients with T2DM and DKD and demonstrated a reduction in urinary protein levels after eight weeks [15]. Similarly, Tiryaki et al. conducted a randomized, placebo-controlled trial including 98 individuals with T2DM supplemented with 0.25 μg/day of calcitriol and observed a significant reduction in proteinuria among patients with early stage DKD in the intervention group [17]. Our group performed a longitudinal study including patients with T1DM who received high-dose cholecalciferol supplementation (10,000 IU/day) for twelve weeks and showed an improvement in UACR in participants who achieved serum 25(OH)D concentrations above 30 ng/mL [14]. In contrast, Das et al., in a study including 100 patients with T2DM and DKD treated with 60,000 IU of cholecalciferol weekly for eight weeks, did not observe significant improvements in renal function following supplementation [16]. Finally, two recent meta-analyses comprising a total of 651 and 734 patients, conducted by Xuan et al. and Gupta et al., respectively, reported a potential benefit of VD supplementation in patients with DKD [18,19]. Our findings, analyzing data from T2DM patients in early and advanced stage of DKD, suggest that a level below 30 ng/mL may be associated with increasing UACR, and if confirmed by future studies and larger samples, could represent a good standard to achieve in clinical practice. Nevertheless, this association was observed only in advanced stages of DKD, and the proposed ≥30 ng/mL threshold remains hypothesis-generating—warranting confirmation in longitudinal and interventional studies.

The reduction in VD concentrations in patients with advanced-stage DKD can be explained by the decreased bioavailability of 25(OH)D_3_ during the progression of kidney disease. The decrease in GFR limits the supply of 25(OH)D_3_ to the 1-α-hydroxylase enzyme in the proximal renal tubule, leading to a decrease in the renal production capacity of 1,25(OH)_2_D [22,23]. Additionally, as the glomerular filtration rate decreases, there is a decrease in the vitamin D-binding protein in the proximal tubule, thereby reducing the tubular reabsorption of 25(OH)D and causing a decrease in both the active and inactive forms of VD [27,59,60]. In CKD, hypovitaminosis D arises through mechanisms related to low concentrations of megalin and cytochrome P 27B1 (CYP27B1) [26]. The decrease in megalin reduces the reabsorption of vitamin D-binding protein and, consequently, the conversion of 25(OH)D_3_ to 1,25(OH)_2_D [61]. The decline in CYP27B1, resulting from proteinuria, decreases the production of 1,25(OH)_2_D_3_ due to the loss of the 1,25(OH)_2_D_3_ complex and vitamin D-binding protein. In addition, the uptake of 25(OH)D_3_ decreases due to the negative regulation of megalin levels [62,63]. Previous studies indicate that VD may act as a nephroprotective factor in patients with diabetes, possibly through mechanisms associated with the inhibition of the renin–angiotensin system, reduction in inflammatory mediators, modulations of nitric oxide, and fibrosis [22,23,24,25]. Vitamin D, therefore, may also exert anti-inflammatory properties due to its antioxidant activity at the renal level, reducing oxidative stress [23,24].

Secondary hyperparathyroidism develops as kidney disease progresses [64,65] and is defined by elevated parathyroid hormone (PTH) concentrations, leading to bone, mineral [50,66], and cardiovascular complications that contribute to increased morbidity and mortality in CKD [67,68]. Sustained high levels of PTH cause nephrotoxicity and accelerate the progression of kidney damage [64,69]. At the same time, reduced VD concentration exacerbates secondary hyperparathyroidism by limiting the renal production of 1,25-dihydroxyvitamin D (1,25D), thereby increasing PTH secretion [70,71]. Some studies have observed the beneficial effects of VD in controlling secondary hyperparathyroidism and consequently CKD progression [72,73,74,75,76].

Several clinical parameters are involved in the progression of CKD including glycemic control, hypertension, use of nephrotoxic drugs, obesity, smoking, and ethnicity [2,77,78]. In this context, although the regression model in our study (R^2^ = 0.103) indicates that the serum VD concentrations explain only about 10% of the variability in the UACR, this value is still relevant. Even with control of the clinical parameters listed above and with well-established treatment strategies, including the use of renoprotective drugs such as GLP-1, SGLT2 inhibitors, and finerenone, the incidence of progression from DKD to dialysis remains substantial. Therefore, any additional factor that may contribute to slowing renal function decline—even if accounting for only 10% of the variability—should be considered clinically relevant.

The statistical power found in most of our comparisons was >0.8. However, the main limitation of our study is the small sample size, and our data need to be confirmed in a larger study. Due to its cross-sectional design, it is not possible to establish a causal relationship between vitamin D concentrations and DKD, and the possibility of reverse causality cannot be excluded.

Furthermore, physical activity, dietary intake, and sun exposure may represent potential confounding factors. To minimize this effect, diet and physical activity were standardized for all participants according to the ADA guidelines [28]. The healthcare team reinforced nutritional guidance aimed at reducing the consumption of processed foods and foods high in salt, added sugars, and fats, while encouraging whole-food choices and supporting realistic dietary planning with patients. Participants were also advised to reduce sedentary behavior by incorporating walking or other light activities according to their fitness level. Because these factors are difficult to measure objectively, standardized nutritional and lifestyle counseling was implemented to promote behavioral uniformity and reduce inter-individual variability. Although solar radiation in the study region is relatively constant and intense throughout the year, the duration of individual sun exposure was not assessed, which represents a study limitation. Nevertheless, previous studies have shown that sun exposure alone may not be sufficient to overcome vitamin D insufficiency or deficiency [79,80,81]. Furthermore, vitamin D deficiency has been reported even in regions with abundant sunlight [81,82,83], suggesting that factors beyond solar availability may influence vitamin D status.

The main strength of our study was the homogeneity of the study population, which was stratified into different stages of DKD based on specific ranges of the UACR, excluding patients on dialysis or pre-dialysis.

## 5. Conclusions

Higher vitamin D concentrations were associated with reduced UACR only in advanced-stage DKD, particularly when the VD levels were ≥30 ng/mL. Nevertheless, it did not occur in the early stages of kidney disease.

## Figures and Tables

**Table 1:** Clinical and demographic characteristics of patients in group 1 and group 2.

Clinical Characteristics	Group 1	Group 2	*p*
	*n* = 29	*n* = 51	
Age (years)	63.7 ± 8.2	66.1 ± 6.5	0.15
Gender (M/F)	15/14	30/21	0.37
BMI (kg/m^2^)	30.7 ± 4.9	29.9 ± 4.1	0.46
Time since diagnosis of T2DM (years)	9.2 ± 6.1	16.8 ± 8.4	<0.01
Dyslipidemia (yes%)	24 (82.8)	47 (92.2)	0.21
Previous cardiovascular event (yes%)	4 (13.8)	5 (9.8)	0.55
History of previous neuropathy (yes%)	16 (55.2)	47 (92.2)	<0.01
History of retinopathy (yes%)	4 (13.8)	9 (17.6)	0.65
History of smoking (yes%)	11 (37.9)	18 (35.3)	0.91
History of alcoholism (yes%)	12 (41.4)	27 (52.9)	0.14

BMI: body mass index; F: female; T2DM: type 2 diabetes mellitus; M: male.

**Table 2:** Laboratory parameters of patients in group 1 and group 2.

Laboratory Features	Group 1	Group 2	*p*
	*n* = 29	*n* = 51	
25(OH)D (ng/mL)	30.2 ± 8	26.7 ± 10.1	<0.05
FPG (mg/dL)	132 ± 40.2	159.6 ± 61.5	0.03
Glycated hemoglobin (%)	7.9 ± 0.9	8.2 ± 1.4	0.27
Creatinine (mg/dL)	0.8 ± 0.2	1.5 ± 0.4	<0.01
GFR (mL/min/1.73 m^2^)	88.1 ± 17.1	51 ± 14.4	<0.01
Total cholesterol (mg/dL)	159 ± 45.9	185.1 ± 48.7	0.02
HDL (mg/dL)	40.4 ± 12.3	40 ± 10.5	0.87
LDL (mg/dL)	85.2 ± 35.2	98.9 ± 43.3	0.16
Triglycerides (mg/dL)	170 ± 83.3	231.6 ± 153.0	0.05
Mean albuminuria (mg/g)	104 ± 67	685 ± 814	<0.01
Mean albuminuria (log10)	1.93 ± 0.27	2.6 ± 0.5	<0.01

25(OH)D: 25-hydroxyvitamin D; GFR: glomerular filtration rate; FPG: fasting plasma glucose; HDL: high density lipoprotein; LDL: low density lipoprotein.

**Table 3:** Comparison of laboratorial characteristics between group 1 and group 2 according to the Institute of Medicine.

Parameters	Group 1		*p*	Group 2		*p*
	VD Deficiency	VD Normality		VD Deficiency	VD Normality	
	<20 ng/mL	≥20 ng/mL		<20 ng/mL	≥20 ng/mL	
	*n* = 4	*n* = 25		*n* = 16	*n* = 35	
Alb. (mg/g)	81.8 ± 60.3	107.0 ± 68.5	0.39	937.6 ± 634	569 ± 868	0.01
Alb. (log10)	1.8 ± 0.3	1.9 ± 0.3	0.39	2.8 ± 0.4	2.5 ± 0.5	0.01
Creat. (mg/dL)	0.8 ± 0.1	0.8 ± 0.3	0.92	1.6 ± 0.5	1.4 ± 0.4	0.09
GFR (mL/min/1.73 m^2^)	86.8 ± 12.9	88.4 ± 17.9	0.69	48.7 ± 16.7	52.1 ± 13.4	0.45
FPG (mg/dL)	136 ± 64.9	131 ± 36.8	0.92	158.2 ± 59.2	160.2 ± 63.4	0.68
HbA1c (%)	7.5 ± 0.9	8 ± 0.9	0.31	8.5 ± 1.6	8.1 ± 1.3	0.27

Alb.: albuminuria; Creat.: creatinine; GFR: glomerular filtration rate; FPG: fasting plasma glucose; HbA1c: glycated hemoglobin; VD: vitamin D.

**Table 4:** Comparison of laboratory characteristics between group 1 and group 2 according to the Endocrine Society.

Parameters	Group 1			*p*	Group 2			*p*
	VD Deficiency	VD Insufficiency	VD Normality		VD Deficiency	VD Insufficiency	VD Normality	
	<20 ng/mL	20–29.9 ng/mL	≥30 ng/mL		<20 ng/mL	20–29.9 ng/mL	≥30 ng/mL	
	*n* = 4	*n* = 9	*n* = 16		*n* = 16	*n* = 19	*n* = 16	
Alb. (mg/g)	81.8 ± 60.3	100.4 ± 68.5	110.8 ± 73	0.64	937.6 ± 634	739.8 ± 1138	365.9 ± 268.3	0.02 *
Alb. (log10)	1.8 ± 0.3	1.9 ± 0.27	2 ± 0.3	0.73	2.8 ± 0.4	2.5 ± 0.5	2.4 ± 0.4	0.04 *
Creat. (mg/dL)	0.8 ± 0.1	0.7 ± 0.2	0.9 ± 0.3	0.13	1.6 ± 0.5	1.4 ± 0.5	1.4 ± 0.3	0.28
GFR (mL/min/1.73 m^2^)	86.8 ± 12.9	96.6 ± 15.6	83.7 ± 17.9	0.20	48.7 ± 16.7	52.1 ± 15	52 ± 11.6	0.75
FPG (mg/dL)	136 ± 64.9	133 ± 45	131 ± 32.9	0.98	158.2 ± 59.2	172.4 ± 59.7	145.7 ± 66.5	0.26
HbA1c (%)	7.5 ± 0.9	8 ± 0.8	8 ± 0.9	0.65	8.5 ± 1.6	8.4 ± 1.4	7.8 ± 1.1	0.23

*: *p* < 0.05; difference between VD normality vs. VD deficiency; Alb.: albuminuria; Creat.: creatinine; GFR: glomerular filtration rate; FPG: fasting plasma glucose; HbA1c: glycated hemoglobin; VD: vitamin D.

**Table 5:** Vitamin D concentrations according to UACR tertiles for patients in group 1 and group 2.

Parameters	Group 1			*p*	Group 2			*p*
	1st Tertile (1.47–1.82)	2nd Tertile (1.83–2.05)	3rd Tertile (2.06–2.46)		1st Tertile (1.63–2.4)	2nd Tertile (2.41–2.84)	3rd Tertile (2.85–3.68)	
	*n* = 10	*n* = 9	*n* = 10		*n* = 17	*n* = 17	*n* = 17	
25(OH)D (ng/mL)	28.4 ± 6.7	31.4 ± 7.9	30.9 ± 9.6	0.69	30 ± 8.5	27.8 ± 9	22.4 ± 11.3	0.01 *

*: *p* < 0.05; difference between 1st tertile and 3rd tertile; 25(OH)D: 25-hydroxyvitamin D.

## Data Availability

The data presented in this study are available on request from the corresponding author due to privacy reasons.

## Abbreviations

The following abbreviations are used in this manuscript:

DKD	Diabetic kidney disease
DM	Diabetes mellitus
VD	Vitamin D
UACR	Urine albumin–creatinine ratio
GFR	Glomerular filtration rate
CKD	Chronic kidney disease
T2DM	Type 2 diabetes mellitus
ICF	Informed consent form
ADA	American Diabetes Association
ACE	Angiotensin-converting enzyme
ARBs	Angiotensin receptor blockers
DEQAS	Vitamin D External Quality Assessment Service
25(OH)D	25-hydroxyvitamin D
HbA1C	Hemoglobin A1C
HPLC	High-performance liquid chromatography
LDL	Low-density lipoprotein
HDL	High-density lipoprotein
IOM	Institute of Medicine
SPSS	Statistical Package for the Social Sciences
ANOVA	One-way analysis of variance
ACEis	Angiotensin-converting enzyme inhibitors
ARBs	Angiotensin ii receptor blockers
SGLT2	Sodium-glucose cotransporter-2
GLP-1	Glucagon-like peptide-1
FPG	Fasting plasma glucose
T1DM	Type 1 diabetes mellitus
PTH	Parathyroid hormone
